# Seizures and Interictal Epileptiform Activity in the Rat Collagenase Model for Intracerebral Hemorrhage

**DOI:** 10.3389/fnins.2021.682036

**Published:** 2021-06-18

**Authors:** Charlotte Germonpré, Silke Proesmans, Charlotte Bouckaert, Mathieu Sprengers, Paul Boon, Robrecht Raedt, Veerle De Herdt

**Affiliations:** 4BRAIN, Department of Neurology, Ghent University, Ghent, Belgium

**Keywords:** intracerebral hemorrhage, stroke, epilepsy, video-EEG monitoring, unprovoked seizures, acute symptomatic seizures, rat collagenase model

## Abstract

**Aims:**

Intracerebral hemorrhage (ICH) is a known risk factor for the development of acute symptomatic as well as late unprovoked seizures. The underlying pathophysiology of post-ICH seizures is incompletely understood and there are no reliable predictive biomarkers. An animal model to study post-ICH seizures is currently lacking. The aim of this study was to investigate (1) the occurrence of seizures and interictal epileptiform activity in the ICH rat collagenase model using long-term video-EEG monitoring (VEM) and (2) whether seizure occurrence was associated with interictal epileptiform activity and histological features.

**Methods:**

Male Sprague-Dawley rats were implanted with epidural electrodes. After 1 week of baseline VEM, collagenase was injected in left striatum to induce an ICH. VEM was continued for 180 days to assess the occurrence of post-ICH seizures and interictal epileptiform activity (spikes and epileptiform discharges). At the end of the experiment, animals were euthanized for histological characterization of the hemorrhagic lesion, using cresyl violet, Prussian blue and immunofluorescence staining.

**Results:**

Acute symptomatic seizures occurred in 4/12 animals between 46 and 80 h after ICH induction. Late unprovoked seizures were present in 2/12 animals and started at 90 and 103 days post-ICH. Animals with late unprovoked seizures did not have acute symptomatic seizures. All electrographic seizures were accompanied by clear behavioral changes. Interictal spikes and epileptiform discharges were observed in all animals but occurred more frequently in rats with late seizures (*p* = 0.019 and *p* < 0.001, respectively). Animals with acute symptomatic seizures had more extended hemorrhagic lesions and hemosiderin deposits in the piriform cortex.

**Conclusion:**

Both acute symptomatic and late unprovoked seizures were observed in the rat collagenase model. Interictal epileptiform activity was more frequently seen in animals with late seizures. Rats with acute symptomatic seizures showed more extensive lesions and hemosiderin deposits in the piriform cortex. This model could be used to further explore possible biomarkers for epileptogenesis.

## Introduction

Spontaneous non-traumatic intracerebral hemorrhage (ICH) is the second most common type of stroke, after ischemic stroke, and occurs in 10–20% of all stroke patients. It is a devastating acute cerebrovascular disorder with high mortality and morbidity. Only 16–25% of surviving patients achieve functional independence (Poon et al., 2014). ICH is also a known risk factor for the development of epileptic seizures, which are a substantial burden for the patient as well as for the healthcare and social care systems (Sen et al., 2020). Moreover, there is evidence that epilepsy negatively influences long-term stroke outcome (Rossi et al., 2013; Madžar et al., 2014).

Early or acute symptomatic seizures are defined as seizures during the first 7 days following stroke and occur in 8–14% of the patients with ICH (De Herdt et al., 2011; Haapaniemi et al., 2014; de Greef et al., 2015). Studies using continuous EEG monitoring have even reported incidences of up to 28–31% (Vespa et al., 2003; Claassen et al., 2007). Acute symptomatic seizures are distinguished from late or unprovoked seizures, which occur later than 7 days post-stroke. This type of seizures is also referred to as post-stroke epilepsy and has a reported incidence of up to 10%, with a median time to first seizure of 6–9 months post-ICH (Rossi et al., 2013; Haapaniemi et al., 2014).

The underlying pathophysiology of acute and late seizures remains largely hypothetical but could involve (1) a direct mechanical effect on the brain tissue by the hemorrhage, (2) transient increases of neuronal excitability caused by blood products, such as thrombin, which induce cellular biochemical dysfunction, and (3) persistent changes in neuronal excitability due to hemosiderin deposits and post-ICH inflammation and gliosis (Willmore et al., 1978; Rosen and Frumin, 1979; Silverman et al., 2002; Xi et al., 2006; Vezzani et al., 2013; Chen et al., 2014; Biffi et al., 2016; Ferlazzo et al., 2016).

There are currently no reliable predictive biomarkers for post-ICH seizures, although the occurrence of acute symptomatic seizures seems to be a risk factor for the development of post-ICH epilepsy (Haapaniemi et al., 2014; Lahti et al., 2017). Besides, continuous EEG monitoring reveals interictal epileptiform abnormalities which are associated with an increased risk of acute and late post-stroke seizures (Mecarelli et al., 2011; Bentes et al., 2018; Chen et al., 2021) and cortical involvement of the hemorrhage has also been associated with the occurrence of both acute symptomatic and late unprovoked seizures (Yang et al., 2009; De Herdt et al., 2011; Rossi et al., 2013).

Preclinical research on post-ICH seizures is needed to further unravel the pathophysiology of post-ICH seizures and to identify possible biomarkers. However, an animal model to study post-ICH seizures is currently lacking. Although intracerebral injections of blood products, such as iron or thrombin, result in epileptic seizures in the acute phase, no hemorrhage is induced (Willmore et al., 1978; Lee et al., 1997). Consequently, other models are more suitable to mimic the human pathophysiology, such as the autologous blood model and the collagenase model. Both the injection of autologous blood (Bullock et al., 1984) and collagenase, an enzyme that dismantles collagen in the basal lamina of the cerebral blood vessels (Rosenberg et al., 1990), reproducibly results in an ICH at the site of injection. One study assessed the occurrence of acute seizures in both models and could only demonstrate seizures after collagenase injection (Klahr et al., 2015). We confirmed this in our previous study and additionally performed video-EEG to characterize the behavioral aspects of the seizures. We found that 45% of rats injected with collagenase in the striatum display acute symptomatic seizures during the first 4 days after the bleeding (Germonpré et al., 2020). However, it is not known if late, unprovoked seizures occur in the rat collagenase model as long-term video-EEG monitoring beyond 30 days post-ICH has not been performed. Moreover, no studies assessed the potential value of interictal epileptiform discharges or histological features of the bleeding as a biomarker for post-ICH seizures.

The aim of the present study was to assess the occurrence of clinical and electrographic post-ICH seizures in the rat collagenase model over a 6-months period using long term video-EEG monitoring. Based on the proposed mechanisms regarding the pathophysiology and biomarkers of post-ICH seizures, the secondary aim of the present study was to evaluate interictal epileptiform activity and histological parameters, such as lesion volume, cortical involvement, hemosiderin deposits and glial markers, as possible biomarkers for post-ICH seizures. Furthermore, long-term functional deficits following ICH and the association with seizure occurrence was studied.

## Materials and Methods

### Animals

**FIGURE 1:**
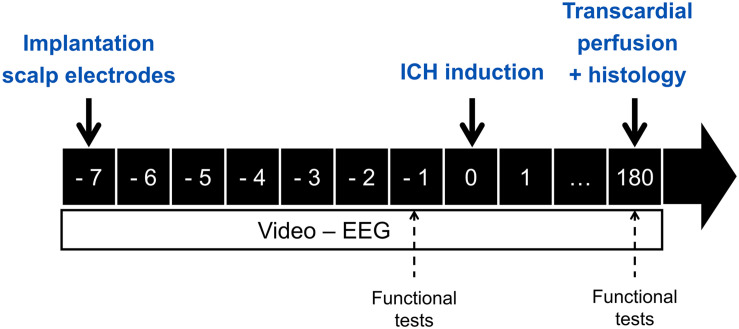
Overview of the timing (in days) of the experimental procedures.

Sixteen male Sprague Dawley rats, weighing 314 ± 3 g, were obtained from Envigo (The Netherlands). Three of these animals were excluded because of implantation related brain damage. Animals were kept under controlled environmental conditions with a 12 h/12 h light-dark cycle, 20–23°C room temperature, 40–60% relative humidity and food and water *ad libitum*. All experimental procedures were in accordance to the European Directive (2010/63/EU) and approved by the local experimental animal committee (Ghent University Hospital, ECD 16/51). A timeline of the experiment is shown in Figure 1.

### Surgery and ICH Induction

Rats were anesthetized with a mixture of medical oxygen and isoflurane (5% for induction, 2% for maintenance) and were placed in a stereotaxic frame. Body temperature was maintained by a heating pad with feedback control using a rectal probe. Epidural recording electrodes were implanted in the os frontale (4.0 AP, ± 2.0 ML) and os parietale (−4.5 AP, ± 3.0 ML). In addition, one electrode was placed in the os occipitale and used as ground/reference electrode. Four anchor screws were placed to secure the head cap. The craniotomy (0.5 AP, 3.5 ML) for later injection of collagenase was made. At the end of the surgery, the connector pins of all electrodes were assembled in a connector block and secured to the skull using metabond (Super-Bond C&B, Sunmedical, Japan) and dental cement (Simplex Rapid fluid/powder, Kemdent, United Kingdom). Craniotomy and bregma were temporarily covered with bone wax and Kwik-Sil (World Precision Instruments, United Kingdom), respectively, until collagenase injection. Buprenorphine (0.3 mg/kg) was administered subcutaneously at the end of the surgery and animals were connected to the video-EEG setup for baseline video-EEG monitoring. After 1 week, the intracerebral hemorrhage was induced by stereotactic injection of collagenase (type VII-S, C2399, Sigma-Aldrich) in the previously made craniotomy (0.5 AP, 3.5 ML, 6.0 DV). Collagenase (0.6 U dissolved in 0.7 μL saline) was infused over 5 min and the syringe was slowly removed after an additional 5 min, to prevent backflow. This was done using a Neuros-Syringe (model 7001 point style 4, Hamilton) and a Quintessential Stereotaxic Injection system (flowrate 0.14 μL/min, Stoelting, United States). After filling the craniotomy with Kwik-Sil, the remaining part of the skull was covered with dental cement and the animals were reconnected to the setup.

### Video-EEG Monitoring

**FIGURE 2:**
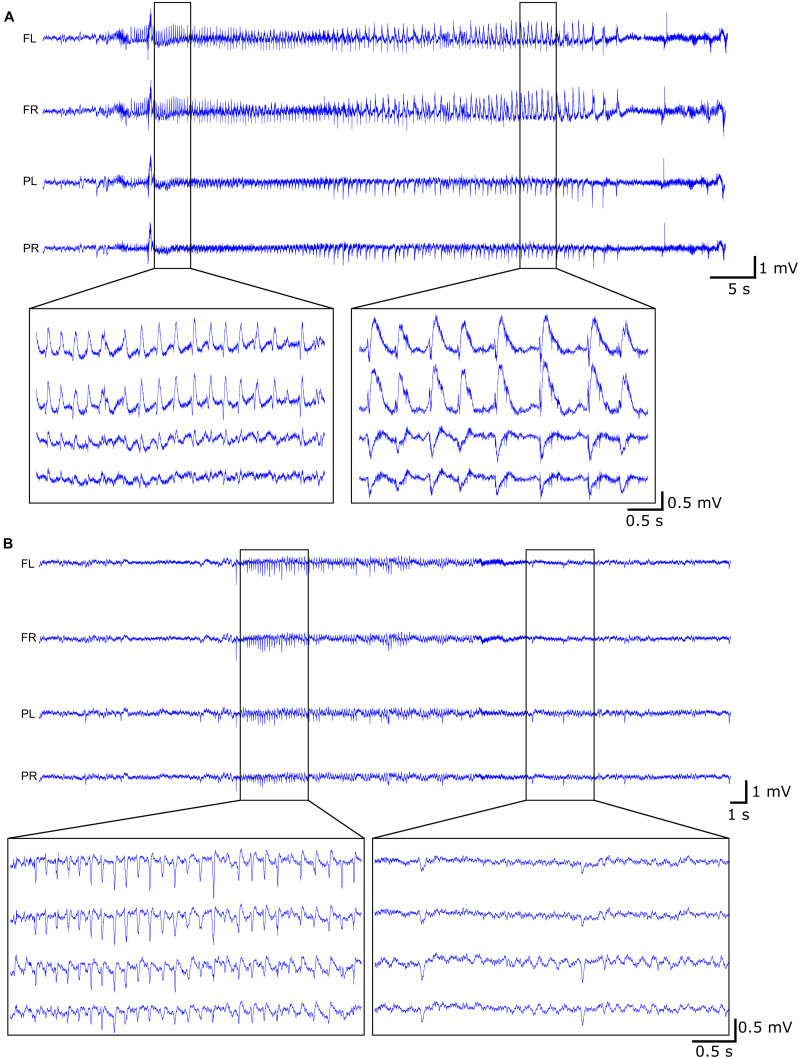
Example of EEG trace during **(A)** late seizure (rat 10) and **(B)** interictal activity (rat 08) with both the epileptiform discharges (left) and the interictal spikes (right).

Continuous video-EEG monitoring (VEM) was performed as previously described (Germonpré et al., 2020). Briefly, the EEG setup consisted of a custom-build head stage, carrying a 4-channel unity gain amplifier, connected via tethers to a 12-channel commutator (Plastics One, Roanoke, VA, United States), allowing the animals to move freely, and a 512× amplifier. The EEG was digitized at a sampling rate of 2 kHz using a NiDAQ card (USB-6259, National Instruments). The animals were placed in plexiglass cages with mirror foil glued to the side and back walls, allowing to visualize the animal from all possible angles, and infrared lights were placed to allow visualization of the animals in the dark. The animals were individually monitored 24/7 by means of a camera (Raspberry Pi 3) placed in front of the cage. A Matlab-based application was used to control the acquisition and storage of the EEG and video-files. The recorded EEG was visually inspected and seizures were annotated. A seizure is defined as a rhythmic pattern of sharp spikes on the EEG, with an amplitude exceeding at least 3 times the baseline amplitude and lasting more than 10 s (Figure 2A). If a seizure was detected on the EEG, the corresponding video-files were used to check the clinical symptoms of the seizure or to exclude non-epileptic activity. The clinical seizures were scored according to the modified Racine Scale (Racine, 1972; Germonpré et al., 2020): (1) wet dog shakes; (2) head nodding, facial clonus, eye blinking, disoriented walk with curved back; (3) unilateral forelimb-clonus; (4) bilateral forelimb-clonus; (5) hindlimb-clonus, rearing, falling. One day per week was screened for interictal epileptiform EEG activity, subdivided in interictal spikes (sharp spikes of less than 100 ms, separated by at least 1.5 s of normal EEG) and epileptiform discharges (cluster of interictal spikes) (Figure 2B). Status epilepticus (SE) was defined as continuous seizure activity for more than 5 min and the end of the SE was defined as the occurrence of inter-spike intervals of at least 1.5 s.

### Functional Testing

Motor deficits following ICH were assessed with the cylinder and forelimb placing test which evaluate forelimb use asymmetry. Both tests were performed 1 day before and 6 months after ICH. The open field test (OFT) was only conducted at the 6 months timepoint. All tests were videotaped and analyzed in a blinded manner.

In the cylinder test rats were placed in a glass cylinder (26 cm diameter, 65 cm height) for 5 min and spontaneous placement of forepaws against the cylinder wall was monitored. The results are expressed as the number of wall touches with the right forepaw (contralateral to the lesion) relative to the total number of forepaw touches.

In the forelimb placing test the whiskers of the animal are gently brushed against the corner of the table and as a reaction, the animal places its forelimb (ipsilateral to the stimulated whiskers) on the table top. This is called a successful trial. Both forelimbs are tested 10 times and trials in which the animal is struggling were not counted. The results are expressed as the amount of successful trials of the right forelimb, since animals are 100% successful with their left, unimpaired forelimb.

To assess spontaneous locomotor activity following ICH, an OFT was conducted. The animals were placed in the center of an open field box of 1 m × 1 m × 0.4 m (length × width × height) and were allowed to explore the box during 10 min. Off-line analysis was performed using Optimouse, an open-source Matlab program and different parameters were assessed: distance traveled, average speed, rearing and fraction of time spent in central zone (50 × 50 cm).

### Transcardial Perfusion and Histology

After 180 days of VEM, the animals were injected with an overdose of sodium pentobarbital (200 mg/kg, i.p.) and transcardially perfused with phosphate-buffered saline (PBS) followed by 4% paraformaldehyde (PFA, pH 7.4). After 24 h post-fixation in PFA, the brains were cryoprotected in increasing sucrose solutions (10–20–30%), snap-frozen in isopentane and liquid nitrogen (−196°C) and stored at −80°C. Coronal cryosections (40 μm) of hemorrhage-containing brain tissue were made using a cryostat (Leica, Germany). Every 10th section (every 400 μm) was used for cresyl violet and hemosiderin stain. For each animal three sections were processed with immunofluorescence staining to analyze expression of astrocytic markers GFAP and vimentin and microglial marker Iba1. All slices were digitized using a Pannoramic 250 digital slide scanner (3DHistech, Hungary) at 20× magnification and analyzed using ImageJ.

#### Cresyl Violet Staining

Sections were mounted on a glass slide and consecutively immersed in a 0.3% cresyl violet solution (10 min), distilled water, 96% ethanol (1.5 min), 100% ethanol (5 min, 2×) and xylene (5 min, 2×) and cover slipped using Entellan. The hemorrhage was manually aligned and the volume of the hemorrhage was calculated as follows: average area of the hemorrhage on all slices containing the hemorrhage × 0.4 (0.04 mm slice thickness + 0.36 mm interslice distance) × number of slices containing the hemorrhage. The cortical involvement of the hemorrhage was determined by aligning the part of the hemorrhage extending in the piriform cortex. Ventricle dilatation was calculated as the difference in area of the ipsilateral and contralateral ventricle.

#### Perl’s Prussian Blue Staining for Hemosiderin

After mounting the sections on a glass slide, the slices were rinsed in distilled water. Afterward the slices were immersed in a ferrocyanide solution (2% potassium ferrocyanide and 2% HCl) during 10 min, rinsed three times in distilled water and counterstained with the neutral red solution for 1 min. Slices were then rinsed in distilled water and dehydrated and cleared as described for the cresyl violet stain. Analysis was done using the color deconvolution function in ImageJ and a threshold was applied on the blue channel. The volume of the thresholded area was determined and the volume of the hemosiderin deposits was calculated similarly as described for the cresyl violet stain. The volume of hemosiderin deposits in the piriform cortex was determined by calculating the thresholded area in the piriform cortex only.

#### Immunofluorescent Staining

Sections were rinsed twice in dH_2_O and were treated with 0.5% H_2_O_2_ (30 min) and 1% H_2_O_2_ (60 min) to block endogenous peroxidase activity. After rinsing the slices twice in PBS, slices were transferred to a blocking buffer, containing PBS, 0.4% Fish Skin Gelatin and 0.2% Triton X, for 45 min. Slices were then incubated in primary antibodies [Rabbit anti-Iba1 (1:500, Abcam, Ab178846), rabbit anti-GFAP (1:1,000, DAKO, Z0334) and mouse anti-vimentin (1:250, DAKO, M072501-2)], diluted in blocking buffer, for 1 h at room temperature and overnight at 4°C. The next day, the sections were washed twice in blocking buffer and were incubated for 1 h in goat anti-rabbit Alexa fluor 594 (1:1,000, Abcam, ab15008) or goat anti-mouse Alexa fluor 488 (1:1,000, Abcam, ab150113). Slices were rinsed in PBS (2×) and a nuclear DAPI staining was performed (1 min), followed by washing the slices in PBS. Slices were mounted on a glass slide and cover slipped using Fluoroshield (F6182, Merck). Five regions of interest (ROIs) throughout the ipsilateral brain hemisphere were selected and the MaxEntropy threshold function in ImageJ was applied. The integrated density (the thresholded area multiplied by the mean gray value) for each ROI and subsequently the average over the 5 ROIs was calculated.

### Statistics

Statistical analyses were performed in SPSS for windows (version 26). Values are expressed as mean ± SEM, unless stated otherwise. Statistical significance was set at *p* < 0.05. Normality of the data was assessed by the Shapiro-Wilk test and by visual inspection of the data. The interictal activity (i.e., the number of interictal spikes or epileptiform discharges) was analyzed by a random effects linear mixed model, to accommodate for missing data of rats losing their head cap before the end of the monitoring period. The interictal activity between animals without seizures, with acute seizures and with late seizures (i.e., “group” factor) was analyzed at different time points (EEG monitoring over 26 weeks, i.e., “time” factor). The interictal spikes or epileptiform discharges were used as the dependent variable, subject ID as random factor and group, time and group by time interactions as fixed factors. The covariance structure with the best fit, based on the Akaike’s Information Criterion (AIC), was the heterogenous first order autoregressive model. Bonferroni corrections were used for *post-hoc* analysis. All obtained data during functional testing and histological analysis were continuous and analyzed accordingly. Statistical analysis for functional testing was performed by an Independent Samples *t*-test (cylinder test and OFT) or a Mann-Whitney *U*-test (forelimb placing test) to compare 2 groups (animals with and without acute symptomatic seizures). Kruskal-Wallis tests were used to compare histological variables (lesion volume and integrated density of immunofluorescent markers) between 3 groups of animals (rats without seizures, rats with acute seizures only, rats with only late seizures). The Spearman correlation for not normally distributed data was used to calculate correlation coefficients between seizure characteristics, histological and functional outcomes.

## Results

### EEG

All animals underwent baseline VEM for 7–10 days, prior to ICH induction. None of the animals had epileptic seizures or epileptic spikes during this period.

**FIGURE 3:**
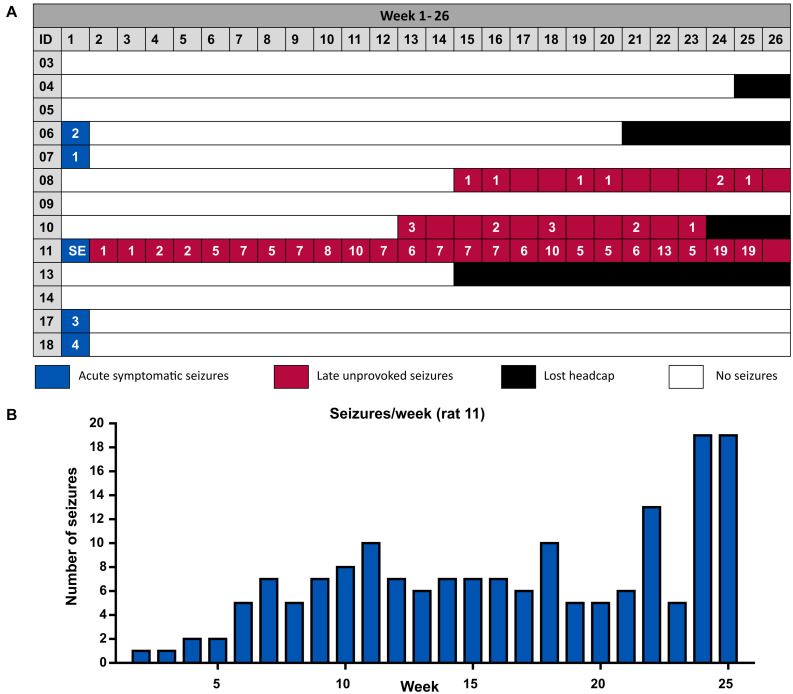
**(A)** Overview of the occurrence of acute symptomatic seizures and late unprovoked seizures per animal. Four animals did not complete the complete monitoring period due to the loss of their head cap with EEG electrodes. The last week of the experiment (week 26) consists of 5 days instead of 7, since monitoring was stopped at 180 days. **(B)** Number of seizures per week of the SE animal.

Acute symptomatic seizures were observed in 4 out of 12 animals (33%) between the 2nd and 4th day after stroke induction (Table 1 and Figure 3A). Mean duration of acute symptomatic seizures was 126 ± 29 s. All acute seizures were accompanied by behavioral changes with a median Racine score of 4 (range 3–5). Late seizures were present in 2 out of 12 animals (17%) and started at 90 and 103 days post-ICH. The animals with late seizures had 0–3 seizures per week and 7 and 11 late seizures in total. The late seizures lasted for 83 ± 17 s and they were all accompanied by behavioral changes with a median Racine score of 4.5 (range 2–5). No significant difference in seizure duration or Racine scale could be observed between the acute and late seizures (Mann-Whitney *U*-test, *p* = 0.121 and *p* = 0.832, respectively). None of the rats with acute seizures had late seizures and vice versa. One animal had a status epilepticus and will be discussed separately.

**TABLE 1:** Characteristics of seizures are visualized. For each animal, the incidence, duration, seizure onset and Racine scores are given.

Rat	Incidence	Duration (s) (mean ± SEM)	Time of onset after ICH	Racine scores
**Acute symptomatic seizures**				
06	2	104.32 ± 23.61	3 days 7 h 52 min	4, 4
07	1	67.46	2 days 8 h 4 min	3
17	3	41.61 ± 5.56	2 days 1 h 41 min	3, 3, 4
18	4	215.8 ± 35.27	1 day 22 h 23 min	5, 3, 5,5
**Late unprovoked seizures**				
08	7	21.23 ± 1.91	103 days 0 h 46 min	2, 3, 4, 4, 2, 2, 2
10	11	122.22 ± 21.15	90 days 10 h 3 min	2, 4, 5, 5, 5, 5, 5, 5, 5, 5, 5

Interictal spikes and epileptiform discharges were observed in all animals. Interictal spikes were seen throughout the whole monitoring period while the number of epileptiform discharges increased over time. Both the presence of the interictal spikes and the epileptiform discharges were compared for the 3 groups of animals over 26 weeks. An overall significant difference was found for the interictal spikes (group^∗^time, *p* = 0.019) and the epileptiform discharges (group^∗^time, *p* < 0.001). A *post-hoc* test with Bonferroni correction revealed that animals with late seizures had a significantly higher amount of interictal spikes at several timepoints (week 1, 4, 8, 12, and 22) and a higher amount of epileptiform discharges between 6 and 19 weeks after ICH induction, compared to animals with acute or no seizures (Supplementary Figure 1).

### Functional Testing

Four animals were euthanized before the end of the monitoring period due to early loss of the electrode head cap. These animals did not undergo functional testing 180 days post-ICH. Consequently, only 1 animal with late seizures, 3 animals with acute seizures and 4 animals with no seizures could be tested at 6 months, so statistical analysis could only be performed for animals with and without acute seizures.

Compared to baseline, there were no statistically significant differences in forelimb use asymmetry 6 months after ICH in the cylinder test (Paired Samples *T*-Test, *p* = 0.279) and the forelimb placing test (Wilcoxon Signed-Rank test, *p* = 0.18) (Supplementary Figure 2A). Statistically significant differences could not be demonstrated between animals with and without acute seizures in the outcome of the cylinder test (Independent Samples *T*-Test, *p* = 0.584) and forelimb placing test (Mann-Whitney *U*-test, *p* = 1) (Supplementary Figure 2B). Regarding the OFT, time spent in center (Independent Samples *t*-test, *p* = 0.130), distance traveled (Independent Samples *t*-test, *p* = 0.454), average speed (Independent Samples *T*-Test, *p* = 0.491) and rearing (Mann-Whitney *U*-test, *p* = 0.174) were not significantly different between the group of animals with acute seizures and the group of animals without acute seizures. Results for each group are presented in Supplementary Table 2.

### Histology

**FIGURE 4:**
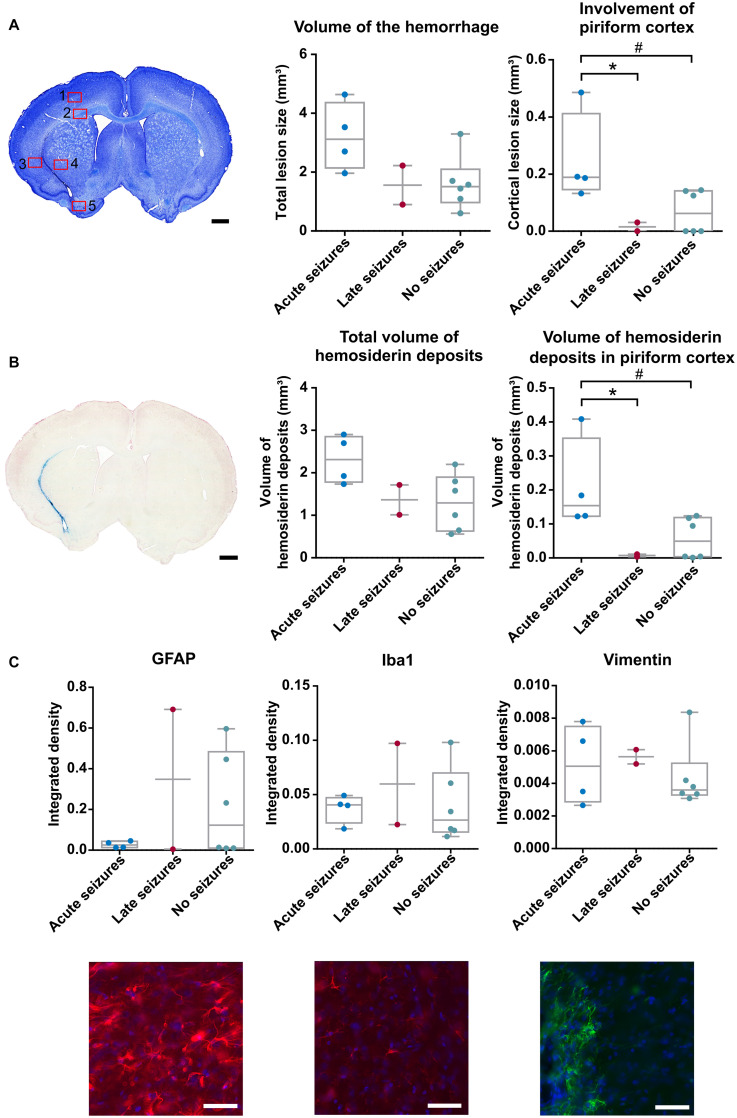
Histology was performed 6 months after ICH induction. **(A)** Cresyl violet staining of an animal with acute symptomatic seizures (rat 07). Red squares represent the ROIs taken for the immunofluorescence staining. A trend toward a significant difference in lesion volume of the hemorrhage was found between the 3 groups of animals [Kruskal-Wallis test, χ^2^(2) = 4.878, *p* = 0.087] whereas the involvement of the piriform cortex was significantly larger in the animals with acute seizures [Kruskal-Wallis test, χ^2^(2) = 6.071, *p* = 0.048; ^#^*p* = 0.032 (adj *p* = 0.095); *p = 0.042 (adj *p* = 0.125)]. **(B)** Perl’s Prussian Blue staining of an animal with acute seizures (rat 07). The volume of hemosiderin deposits did not statistically differ in the total brain [Kruskal-Wallis test, χ^2^(2) = 4.878, *p* = 0.087] but did in the piriform cortex [Kruskal-Wallis, χ^2^(2) = 6.399, *p* = 0.041; ^#^*p* = 0.03 (adj p = 0.089); *p = 0.033 (adj p = 0.1)]. **(C)** Results of the Kruskal-Wallis tests and representative images of the GFAP, IBa1 and Vimentin staining in the striatum in an animal with late seizures (rat 10) [χ^2^(2) = 0.032, *p* = 0.984; χ^2^(2) = 0.651, *p* = 0.722; χ^2^(2) = 0.955, *p* = 0.620, respectively]. Scalebar for A and B is 1 mm, scalebar for C is 50 μm.

Collagenase injection induced an ICH in all animals, with a median lesion volume of 1.83 mm^3^ (range 0.60–4.97 mm^3^, mean 2.20 ± 0.37 mm^3^), measured 6 months after ICH induction on a cresyl violet stain. When the lesion volume was compared between the animals with acute, late and no seizures, a trend toward significance could be detected [Kruskal-Wallis test, χ^2^(2) = 4.878, *p* = 0.087] (Figure 4A). If we more specifically look at the lesion size in the piriform cortex, a significant higher involvement of the piriform cortex was detected in animals with acute seizures compared to the other groups [Kruskal-Wallis test, χ^2^(2) = 6.071, *p* = 0.048]. Similar results were obtained for the Perl’s Prussian blue staining. A trend toward significance was observed with total volume of hemosiderin deposits [Kruskal-Wallis test, χ^2^(2) = 4.878, *p* = 0.087], while the volume of hemosiderin deposits in the piriform cortex was significantly higher in animals with acute symptomatic seizures [Kruskal-Wallis test, χ^2^(2) = 6.399, *p* = 0.041] (Figure 4B), compared to animals with late seizures and no seizures. All values for each group are presented in Supplementary Table 3. The density of GFAP-, vimentin- and Iba1-positive cell bodies and processes was similar between the 3 groups of animals [Kruskal-Wallis test, χ^2^(2) = 0.032, *p* = 0.984; χ^2^(2) = 0.955, *p* = 0.620; and χ^2^(2) = 0.651, *p* = 0.722, respectively] (Figure 4C). In depth analysis of the 5 different ROIs revealed no significant differences between animals with acute, late or no seizures, except for a slight increase of Iba1-positive cells in the piriform cortex [Kruskal-Wallis test, χ^2^(2) = 4.713, *p* = 0.095] and vimentin-positive cells in the motor cortex [Kruskal-Wallis test, χ^2^(2) = 4.851, *p* = 0.088]. The results of the other ROIs can be found in Supplementary Table 4 and Supplementary Figure 3. Ventricle dilatation occurred in all animals: the left (ipsilateral) ventricle was larger than the right (contralateral) ventricle (Paired Samples *T*-Test, *p* < 0.001), but no statistical differences were found when the ventricle dilatation (left—right) was compared between the 3 groups [Kruskal-Wallis test, χ^2^(2) = 2.316, *p* = 0.314].

### Post-ICH status epilepticus

One of the animals (rat 11 on Figure 3A) had a status epilepticus (SE) starting 56 h after collagenase injection and lasting more than 14 h. This SE was behaviorally characterized by repeated wet dog shakes, facial clonus, eye blinking, disoriented walk, bilateral forelimb clonus, rearing, falling and frothing. Late unprovoked seizures in this rat started 13 days after ICH induction (170 seizures in total, distributed over 97 days; mean seizure duration 58.9 ± 1.2 s, number of seizures per week increased over time but seizure duration remained unchanged) (Figure 3B). All seizures were clinical and had a maximum score of 5 according to our modified Racine Scale.

Functional testing was performed at the end of the 180-day monitoring period. The SE animal scored less than the other animals on the cylinder test (SE 32% vs. 41 ± 8%) and the forelimb placing test (SE 0 vs. 9.4 ± 0.4) and was the most active in the OFT (distance traveled SE 760 cm vs. 430 ± 25 cm; average speed SE 1.3 cm/s vs. 0.73 ± 0.04 cm/s; time spent center SE 109 s vs. 96 ± 22 s). All values for each group are presented in Supplementary Table 2.

For histological analysis, lesion volume and cortical involvement as measured by the cresyl violet stain was smaller compared to the other animals (SE 1.72 mm^3^ vs. 2.14 ± 0.35 mm^3^ and SE 0.008 mm^3^ vs. 0.12 ± 0.04 mm^3^, respectively), while there were more hemosiderin deposits in the total brain compared to the other animals (SE 2.27 mm^3^ vs. 1.65 ± 0.22 mm^3^). The volume of the hemosiderin deposits in piriform cortex was smaller (SE 0.031 mm^3^ vs. 0.1 ± 0.03 mm^3^) (Supplementary Table 3). The hemorrhage in this animal extended to the thalamus (mainly in the lateral thalamic nuclei, difficult to localize precisely due to the distortion of the brain).

## Discussion

The present study aimed to assess the occurrence of post-ICH seizures over a prolonged monitoring period. We could demonstrate the occurrence of both acute symptomatic and late unprovoked seizures in the rat collagenase model for ICH. The second aim was to evaluate possible biomarkers for post-ICH seizures. The small number of animals with late seizures prevented us from making strong conclusions about the association between lesion volume or glial markers and the occurrence of seizures but we found that animals with late seizures presented more often with interictal epileptiform activity and rats with acute symptomatic seizures showed more extensive lesions and hemosiderin deposits in the piriform cortex. The rat collagenase model may be suitable to investigate mechanisms underlying post-ICH ictogenesis (i.e., the transition of the interictal state to a seizure) and epileptogenesis (i.e., the transition of a healthy brain to a brain capable of generating spontaneous seizures) but will require the inclusion of a larger number of animals. To our knowledge, no other preclinical studies in an animal model for ICH have investigated the occurrence of both acute and late seizures using long-term continuous VEM.

Acute symptomatic seizures were present in 33% of the animals, which is similar to our previous study in the ICH rat collagenase model where we found an incidence of acute seizures of 45% (Germonpré et al., 2020). This incidence is higher compared to the reported incidence of 8–14% acute symptomatic seizures post-ICH in the human situation (De Herdt et al., 2011; Haapaniemi et al., 2014; de Greef et al., 2015). This difference might be due to the reproducible large lesion size and cortical involvement in the rat model, compared to varying severity and localization in the clinical setting (Germonpré et al., 2020). The lack of continuous EEG monitoring in patients might also result in an underestimation of acute seizure occurrence, since Vespa et al. (2003) showed an incidence of 28% upon continuous EEG monitoring.

Using long-term continuous VEM and a reproducible injection method in our study, late seizures were observed in 17% of the animals, starting 3 months post-ICH. Clinical studies on post-stroke epilepsy report a seizure incidence of up to 10% in ICH (Rossi et al., 2013; Haapaniemi et al., 2014). The incidence of late seizures in our study is comparable to the photothrombosis model for ischemic stroke where an incidence of 18% (7/40 animals) over a period of 2–6 months was reported (Karhunen et al., 2007).

Interictal epileptiform activity could be detected in all animals but was more frequent in animals with late seizures. The fact that all animals showed interictal epileptiform abnormalities could suggest that upon a longer monitoring period, more animals could have developed late seizures. Few research on interictal epileptic activity has been performed in the rat collagenase model. Only two studies report abnormal interictal activity during the first 2 days post-ICH but only in rats with acute symptomatic seizures that were only monitored for 1 week or less (Klahr et al., 2015; Wilkinson et al., 2020). Interictal activity was also observed in studies on ischemic stroke (Kharlamov et al., 2003; Karhunen et al., 2006; Miller et al., 2018). In one study, 35% of the rats developed interictal epileptiform activity over the 12-month monitoring period. Nevertheless, this activity could not be associated with the development of acute or late seizures (Karhunen et al., 2006). Clinical research already showed the role of epileptiform abnormalities in predicting acute post-stroke seizures (Mecarelli et al., 2011) and more recent research also showed an increased risk of late seizures following early epileptiform abnormalities in patients suffering from acute brain injury (Bentes et al., 2018; Punia et al., 2019; Chen et al., 2021). Furthermore, there is some evidence that post-stroke interictal epileptiform activity is associated with neurological deterioration (Claassen et al., 2007; Scoppettuolo et al., 2019).

One of the animals presented with a SE, 3 days after hemorrhage induction. No other preclinical studies reported on the occurrence of SE following collagenase injection. In the clinical setting, post-stroke SE is seen in 1–7% of patients (Beghi et al., 2011; De Herdt et al., 2011). In our animal with SE, late unprovoked seizures started 10 days following the SE with a mean seizure frequency of 1.02 seizures/day. This timing and frequency is different from what we observed in the other animals with late seizures, in whom seizures only started after 3 months and occurred less frequently (0.11 seizures/day). However, this timeframe post-SE is comparable to other post-status epilepticus animal models of epilepsy, for example the intrahippocampal kainic acid model, where spontaneous seizures start 28 ± 4 days after ICH with a median seizure frequency of 0.56 seizures/day (range 0–7.4) (Raedt et al., 2009).

The mean lesion volume of ICH in this experiment (2.2 mm^3^) at 6 months post-ICH is comparable to the residual lesion volume (1.6 mm^3^) at 1 month as reported in the study of Wasserman et al. (Wasserman et al., 2008). These volumes are smaller compared to what we previously reported (26 mm^3^) at 7 days post-ICH (Germonpré et al., 2020), since hematoma resolution occurs over time. After the initial growth during the first days, the hematoma is slowly resolving (Rosenberg et al., 1990; Del Bigio et al., 1996). At 7 days, the hemorrhage volume is equal to the volume at 1 h (Da Silva-Candal et al., 2015). We did not investigate the hematoma evolution during our current experiment.

We observed an association between the occurrence of acute symptomatic seizures and hemorrhagic involvement of the piriform cortex. Patient studies have previously reported an association between the cortical involvement of the hemorrhage and the presence of acute symptomatic seizures (Yang et al., 2009; De Herdt et al., 2011). Some clinical studies also showed an association with late unprovoked seizures (Rossi et al., 2013; Haapaniemi et al., 2014) but we could not demonstrate this in our study. We injected collagenase in the left striatum in close proximity of the piriform cortex, a highly epileptogenic brain region (Vismer et al., 2015), whereas cortical involvement in patients implies all cortical areas where the hemorrhage is extending. We also observed a trend toward an increased lesion volume in animals with acute seizures. This confirms the findings of our previous study, in which we could only detect a trend toward an association between cortical involvement or volume and the presence of acute symptomatic seizures (Germonpré et al., 2020).

The association of the piriform cortical involvement with the presence of acute symptomatic seizures was also detected with the Perl’s Prussian Blue staining for hemosiderin. Animals with acute seizures had a higher volume of hemosiderin deposits in the piriform cortex compared to animals with late or no seizures. Hemosiderin formation following ICH results from erythrocyte lysis and the subsequent breakdown of hemoglobin to heme iron which is then converted to ferritin and hemosiderin (Bradley, 1993; Wagner et al., 2003). However, hemosiderin deposits are only observed 5–6 days following ICH (Koeppen et al., 1995; Wagner et al., 2003) while the acute seizures in this study occur between 2 and 4 days post-ICH. This implies that hemosiderin deposits itself are probably not responsible for the acute symptomatic seizures and are rather related to the volume of the hemorrhage.

Following ICH, an inflammatory response is triggered and activation of microglia can be seen 1 h after collagenase injection (Wang and Doré, 2007). Astrocytes are also activated post-ICH and are enhanced in the perihematomal region (Wang and Doré, 2008; Wang, 2010). Thirty days following ICH, still more astrocytes were seen compared to sham animals (Mestriner et al., 2011). Furthermore, neuroinflammation plays an important role in epileptogenesis (Vezzani et al., 2013). In our study, GFAP, Vimentin and Iba1 expression was observed in the perihematomal region but no association could be found with the incidence of acute or late seizures. However, a trend toward more microglia in the piriform cortex could be detected in animals with acute seizures. Observations in a rat model for ischemic stroke revealed an increase of Vimentin and GFAP in the glial scar but there was no evidence to a correlation with epileptic seizures (Kelly et al., 2018).

Six months following ICH induction, the animals are almost completely recovered and, compared to baseline, no significant functional impairment was detected in the cylinder test and the forelimb placing test. This is line with what other studies reported: no difference between sham and collagenase rats can be demonstrated on the cylinder test and forelimb placing test at 8–10 weeks post-ICH (Hartman et al., 2009; Liu et al., 2013). These results are in contrast to the clinical situation, where only 16–25% of the patients are fully independent at 1 year post-ICH (Poon et al., 2014). However, preclinical evidence suggests that longer-lasting deficits can be observed with more specialized functional testing methods, such as the rotarod test or the catwalk test for gait analysis (Hartman et al., 2009; Liu et al., 2013). No difference on the long-term functional outcome could be demonstrated between animals with and without acute symptomatic, reflecting the almost completely functional recovery 6 months after the ICH induction. Our previous study could demonstrate a difference on the short-term between these 2 groups of animals, in which animals with seizures scored better on the cylinder test (Germonpré et al., 2020).

Our study has some limitations. First, sham animals were not included in this study. Due to the limited amount of monitoring units and the need for long-term availability of the setup, we choose to include a maximum number of collagenase injected animals instead of sham animals. However, we have several arguments to state that the (late) seizures we observed can be attributed to the collagenase-induced ICH. (1) Previous experiments in this model showed that no seizures were induced following saline injection in the acute phase (Germonpré et al., 2020). (2) Studies investigating post-stroke epilepsy in ischemic stroke models included control animals which underwent electrode implantation and no seizures were observed during long-term EEG monitoring (Karhunen et al., 2007; Kelly et al., 2018). (3) Additionally, we tried to minimize the risk of implantation-related seizures by including a baseline monitoring period between electrode implantation and ICH induction. In this way we could confirm the absence of seizures due to electrode implantation. We did not detect seizures in any of the 16 animals during this period. We also excluded animals with pronounced brain damage due to electrode implantation.

Second, only a limited number of animals in our study developed late seizures following ICH, making it harder to investigate the correlation between the occurrence of late seizures and histological features or interictal epileptiform activity. Further research needs to be done to confirm the association between interictal epileptiform abnormalities and late seizures.

We could also not investigate the impact of late seizures on the functional outcome post-ICH, since only 1 animal with late seizures was left to perform behavioral testing.

## Conclusion

We demonstrated the occurrence of acute and late post-ICH seizures in the ICH rat collagenase model using continuous video-EEG monitoring. Acute symptomatic seizures did not seem to predict late seizures. Piriform cortical involvement and hemosiderin deposits were associated with the occurrence of acute symptomatic seizures, whereas animals with late seizures had more epileptiform interictal activity. This model can be used to further investigate post-ICH epileptogenesis and biomarkers for seizures.

## Data Availability Statement

The raw data supporting the conclusions of this article will be made available by the authors, without undue reservation.

## Ethics Statement

The animal study was reviewed and approved by the Ethical Committee on Animal Experiments of Ghent University.

## Author Contributions

CG, MS, PB, RR, and VD contributed to the study design and analysis plan. CG, SP, and CB obtained the data. CG, RR, and VD analyzed the data and prepared the manuscript. All authors reviewed the manuscript.

## Conflict of Interest

The authors declare that the research was conducted in the absence of any commercial or financial relationships that could be construed as a potential conflict of interest.
