# Supplementary material for: Seizures and Interictal Epileptiform Activity in the Rat Collagenase Model for Intracerebral Hemorrhage
Source: Front Neurosci. 2021 Jun 18;15:682036. doi: 10.3389/fnins.2021.682036 (PMC8249930; doi:10.3389/fnins.2021.682036)
Supplement: Supplementary file 1 [file Data_Sheet_1.docx]

Supplementary Material

# Supplementary Figures


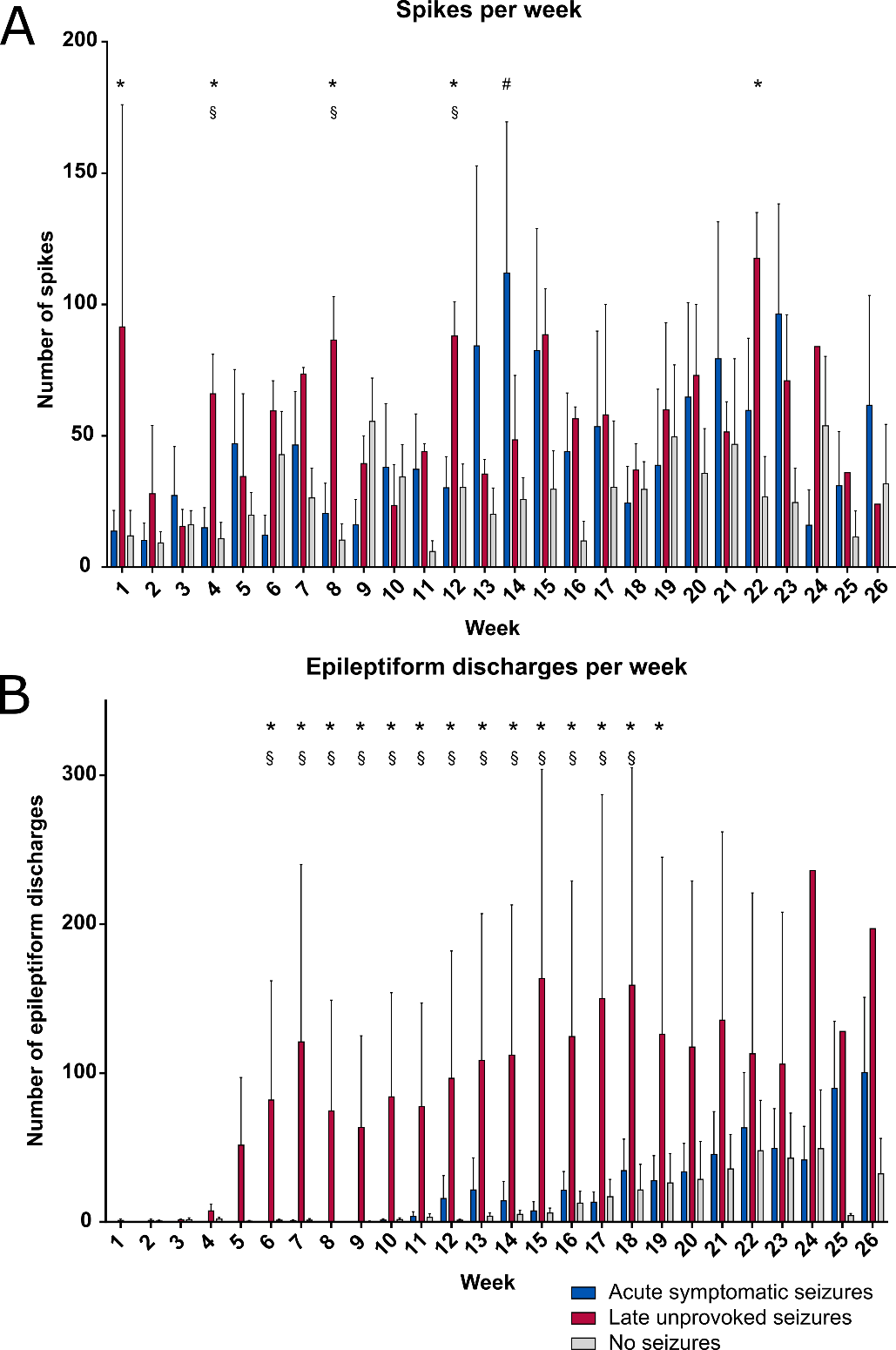


**Supplementary Figure 1.** Overview of the number of **(A)** spikes and **(B)** epileptiform discharges per week following ICH (*p<0.05, late seizures compared to no seizures; ^§^p<0.05, late seizures compared to acute seizures; ^#^p<0.05, acute seizures compared to no seizures).


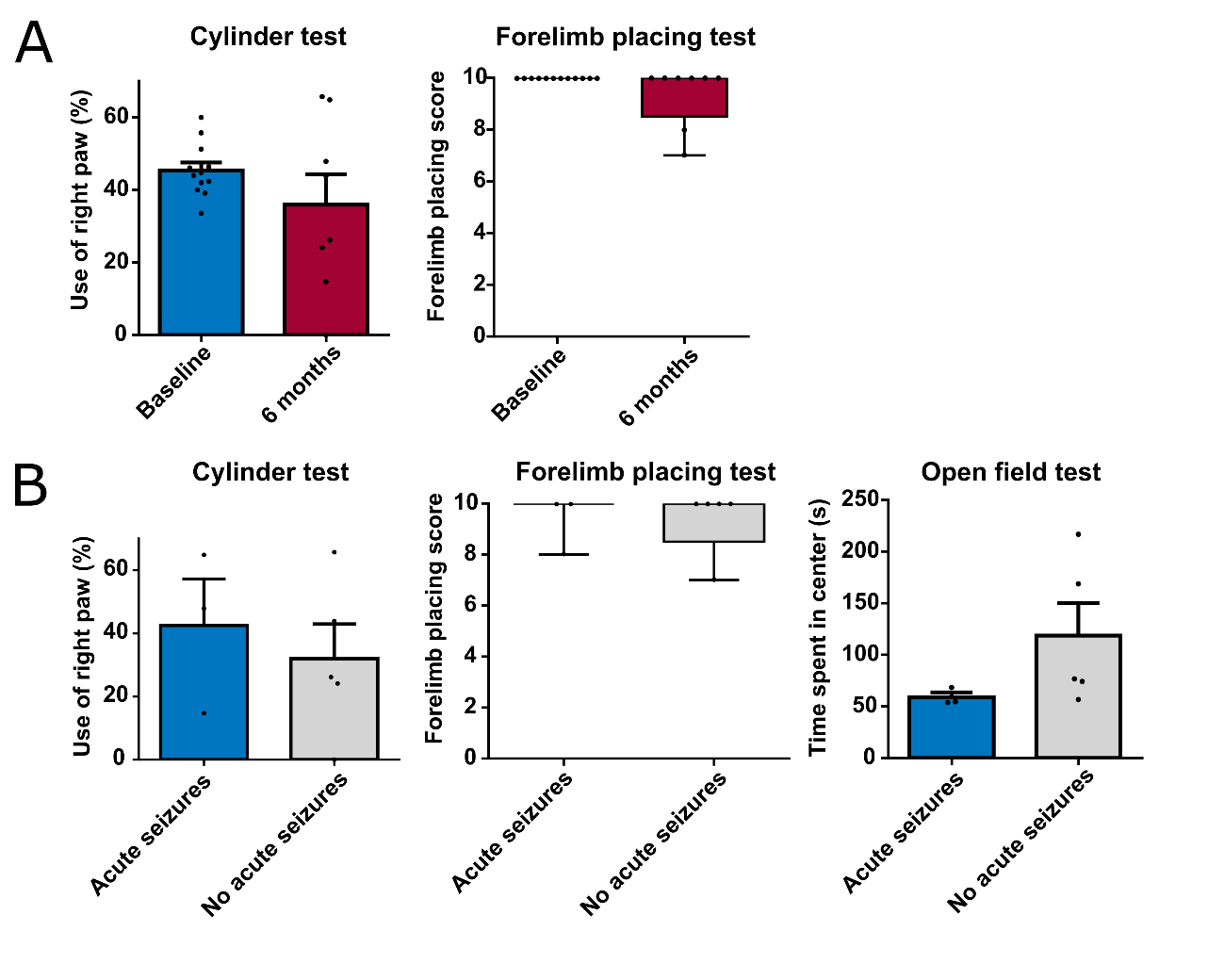


**Supplementary Figure 2: (A)** No statistically significant functional deficits were observed 6 months after ICH induction, compared to baseline. **(B)** No significant difference could be detected at 6 months post-ICH between animals with acute symptomatic seizures and animals with late unprovoked seizures on the cylinder test (Independent Samples T-Test, p=0.536), forelimb placing test (Mann-Whitney U test, p=1.000) and the open field test (Independent Samples T-Test, p=0.335).


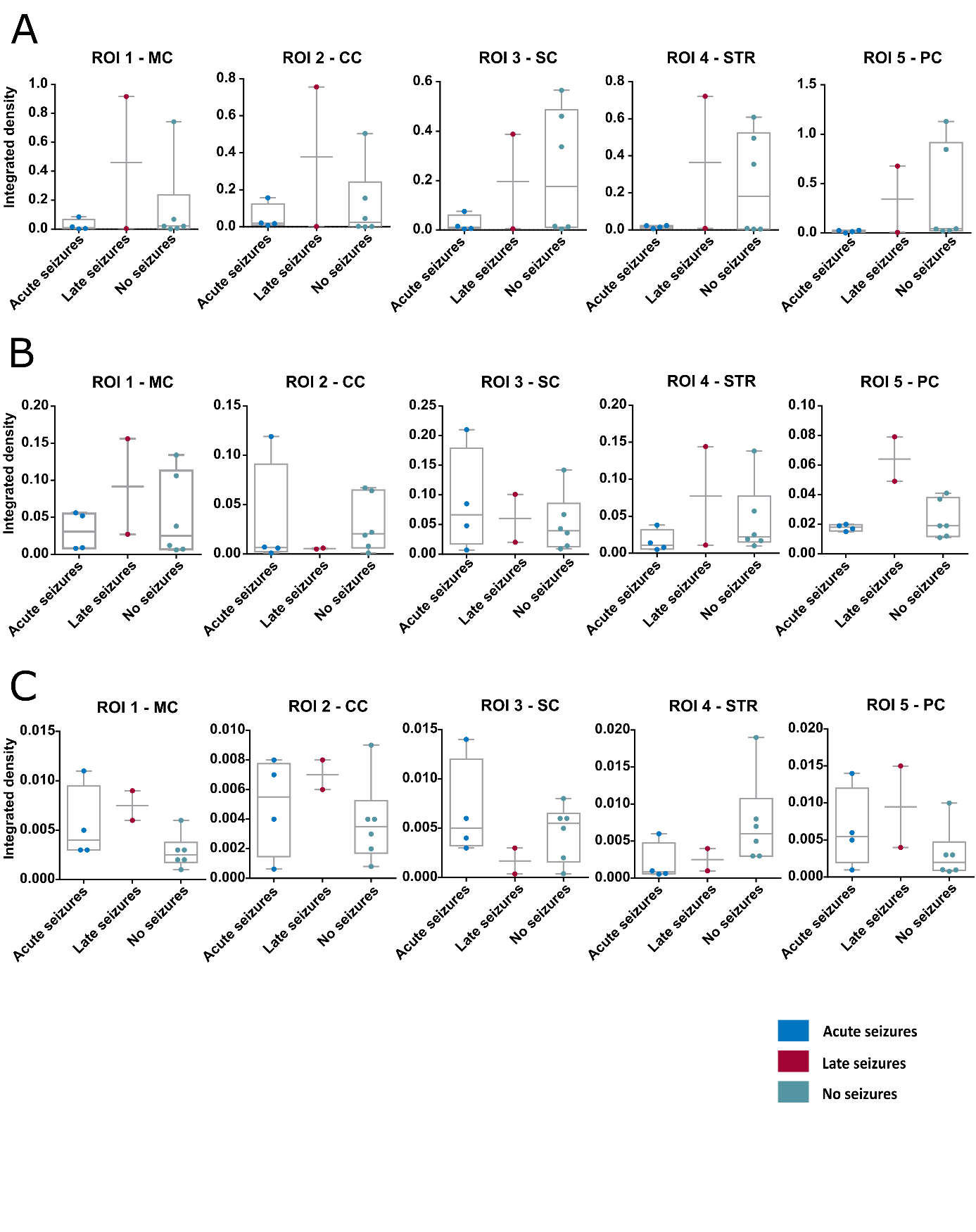


**Supplementary Figure 3:** Results of the Kruskal-Wallis tests for each ROI for (A) GFAP (B) Iba1 and (C) Vimentin. (MC = motor cortex; CC = corpus callosum; SC = somatosensory cortex; STR = striatum; PC = piriform cortex). Results of the statistical tests can be found in table S4.

# Supplementary Tables

**Supplementary Table 1**: Results of the Spearman correlation between seizure characteristics or results of the functional tests and histological parameters. Significant correlations are marked in blue (* p<0.05). Trends are marked in green.

|  | **Lesion volume (CV)** | | **Hemosiderin (total)** | | **Hemosiderin (piriform)** | |
| --- | --- | --- | --- | --- | --- | --- |
|  | r-value | p-value | r-value | p-value | r-value | p-value |
| **Seizure characteristics** | | | | | | |
| Number of seizures | 0.310 | 0.327 | 0.325 | 0.303 | 0.193 | 0.548 |
| Total time in seizure | 0.332 | 0.291 | 0.377 | 0.227 | 0.264 | 0.407 |
| Mean seizure duration | 0.37 | 0.237 | 0.429 | 0.164 | 0.365 | 0.243 |
| Single spikes/week | 0.098 | 0.762 | 0.007 | 0.983 | -0.067 | 0.837 |
| Group spikes/week | -0.028 | 0.931 | -0.168 | 0.602 | 0.014 | 0.965 |
| **Functional testing** | | | | | | |
| Forelimb placing | -0.73 | 0.039 * | -0.733 | 0.039 * | -0.031 | 0.941 |
| Cylinder | -0.6 | 0.115 | -0.681 | 0.063 | -0.275 | 0.509 |
| Time in center OFT | 0.197 | 0.641 | 0.08 | 0.85 | -0.850 | 0.007 * |
| Distance OFT | -0.016 | 0.97 | -0.095 | 0.823 | 0.287 | 0.49 |
| Average speed OFT | -0.032 | 0.941 | -0.109 | 0.797 | 0.287 | 0.49 |
| Rearing OFT | -0.265 | 0.526 | 0.0 | 1 | 0.139 | 0.742 |

**Supplementary Table 2**: Results of the different behavioral tests, for each group of animals (mean ± SEM).

|  | **No seizures (n=4)** | **Acute seizures (n=3)** | **Late seizures (n=1)** | **Status Epilepticus (n=1)** |
| --- | --- | --- | --- | --- |
| **Cylinder Test** | | | | |
| Use of right paw (%) | 29.01 ± 13.60 | 42.48 ± 14.73 | 43.90 | 31.73 |
| **Forelimb placing test** | | | | |
| Forelimb placing score | 9.25 ± 0.75 | 9.33 ± 0.67 | 10 | 0 |
| **Open Field Test** | | | | |
| Time spent in center (s) | 106.08 ± 37.14 | 59.011 ± 4.73 | 168.90 | 109.17 |
| Distance (cm) | 407.95 ± 29.72 | 475.29 ± 58.42 | 441.11 | 760.39 |
| Average Speed (cm/s) | 0.69 ± 0.05 | 0.77 ± 0.10 | 0.74 | 1.30 |
| Rearing | 9.75 ± 2.75 | 5.67 ± 3.67 | 4 | 3 |

**Supplementary Table 3:** Results of the histological assessment, for each group of animals (mean ± SEM).

|  | **No seizures**  **(n=6)** | **Acute seizures**  **(n=4)** | **Late seizures**  **(n=2)** | **Status Epilepticus**  **(n=1)** |
| --- | --- | --- | --- | --- |
| **Cresyl violet** | | | | |
| Lesion volume (mm³) | 1.62 ± 0.37 | 3.21± 0.58 | 1.56 ± 0.66 | 1.72 |
| Cortical involvement (mm³) | 0.07 ± 0.03 | 0.25 ± 0.08 | 0.02 ± 0.02 | 0.008 |
| **Hemosiderin deposits** | | | | |
| Total (mm³) | 1.30 ± 0.27 | 2.32 ± 0.29 | 1.36 ± 0.35 | 2.27 |
| Piriform cortex (mm³) | 0.06 ± 0.02 | 0.21 ± 0.07 | 0.007 ± 0.004 | 0.03 |

**Supplementary Table 4**: Results of the Kruskal-Wallis test of the 5 different ROIs for each immunofluorescent antibody. Trends towards significance are marked in green. (MC = motor cortex; CC = corpus callosum; SC = somatosensory cortex; STR = striatum; PC = piriform cortex)

|  | **ROI 1 – MC** | **ROI 2 – CC** | **ROI 3 – SC** | **ROI 4 – STR** | **ROI 5 – PC** |
| --- | --- | --- | --- | --- | --- |
| **GFAP** | χ²(2)=0.686, p=0.710 | χ²(2)=0.322, p=0.851 | χ²(2)=1.162, p=0.559 | χ²(2)=0.581, p=0.748 | χ²(2)=3.199, p=0.202 |
| **Iba1** | χ²(2)=1.154, p=0.562 | χ²(2)=1.975, p=0.372 | χ²(2)=0.417, p=0.812 | χ²(2)=2.365, p=0.306 | χ²(2)=4.713, p=0.095 |
| **Vimentin** | χ²(2)=4.851, p=0.088 | χ²(2)=1.659, p=0.436 | χ²(2)=2.846, p=0.241 | χ²(2)=4.559, p=0.102 | χ²(2)=3.429, p=0.180 |
